# Cytokine polymorphisms in patients with autoimmune hemolytic anemia

**DOI:** 10.3389/fimmu.2023.1221582

**Published:** 2023-11-10

**Authors:** Anna Zaninoni, Bruno Fattizzo, Loredana Pettine, Cristina Vercellati, Anna P. Marcello, Wilma Barcellini

**Affiliations:** ^1^ SC Ematologia, Fondazione IRCCS Ca’ Granda Ospedale Maggiore Policlinico, Milan, Italy; ^2^ Dipartimento di Oncologia e Oncoematologia, Università degli Studi di Milano, Milan, Italy

**Keywords:** warm autoimmune hemolytic anemia, cold agglutinin disease, cytokine polymorphism, interleukin (IL)-6, IL-10, Interferon (IFN)-γ, tumor necrosis factor (TNF)-α, transforming growth factor (TGF)-β

## Abstract

Autoimmune hemolytic anemia (AIHA) is due to autoantibodies with or without complement activation and involves cellular and cytokine dysregulation. Here, we investigated cytokine single-nucleotide polymorphisms (SNPs) of TNF-α, TGF-β1, IL-10, IL-6, and IFN-γ, along with their serum levels. The former were related to hematological parameters, therapy, and clinical outcome. The study included 123 consecutive patients with primary AIHA [77 warm AIHA and 46 cold agglutinin disease (CAD)], followed up for a median of 49 months. Results show that the allelic frequency of TNF-α -308 G/A polymorphisms was significantly lower in patients versus controls. Moreover, the genotypic frequency of TNF-α -308G/A and TGF-β gene codon 25 G/C genotypes was significantly lower in patients versus controls. Considering cytokine SNP genotypes associated with different gene expression levels, TNF-α high gene expression was significantly more frequent in patients, TGF-β and IL-10 high gene expression was higher in patients with more severe anemia, and TGF-β high gene expression was higher in patients with active disease. Considering treatment, TNF-α and TGF-β high gene expression was more frequent in multitreated patients and particularly in CAD. It may be speculated that this genetic predisposition to a stronger inflammatory response may result in a greater immune dysregulation and in a relapsed/refractory disease. Regarding cytokine serum levels, TNF-α and TGF-β were significantly lower, and IL-10 and IL-6 were significantly higher in patients versus controls, underlying the complex interplay between genetic background and disease features.

## Introduction

Autoimmune hemolytic anemia (AIHA) is characterized by the increased destruction of red blood cells (RBCs) by anti-RBC autoantibodies, with or without complement activation. According to the autoantibody Ig class and thermal characteristics (thermal range of reaction with autologous erythrocytes), AIHAs can be classified as warm (wAIHA), cold [cold agglutinin disease (CAD)], mixed, and atypical forms (1–3).

The pathogenesis of AIHA is complex and involves autoantibodies, monocyte/macrophages, complement, and dysregulation of humoral immunity (B-lymphocytes and long-lived plasma cells) as well as T cells (2, 4–6). The latter include cytotoxic, helper, and regulatory subsets. In particular, T regulatory cells (Treg) are key players in immunologic tolerance, producing interleukin (IL)-10 and transforming growth factor β (TGF-β). Moreover, the IL-10/IL-12 disequilibrium as well as the altered T helper 17/Treg ratio may play a role in the onset and/or maintenance of autoimmunity (2). Many other abnormalities of immunoregulatory cytokines have been described in serum and culture experiments in AIHA (7–9). However, cytokine levels are affected by several incidental factors, including overt and subclinical infections, inflammatory triggers, comorbidities, and AIHA clinical phases and therapy. These confounders may alter the interpretation of cytokine landscape in the pathogenesis of AIHA. There is growing interest in the possible role of the genetic background in the regulation of immune effectors and cytokines in several autoimmune diseases, including outcome and response to biological drugs (10–14). Specifically, single-nucleotide polymorphisms (SNPs) in the regulatory sequences of cytokine genes may provide further insights into cytokine patterns beyond incidental confounders.

We investigated cytokine SNPs of several cytokines, including TNF-α promoter, TGF-β1 gene codon, IL-10 promoter, IL-6 promoter, and IFN-γ genes, along with their serum levels, in AIHA. The results were related to hematological parameters, therapy, and clinical outcome.

## Methods

### Study population

The study population included 123 consecutive patients with primary AIHA, enrolled from 2001 to 2021 diagnosed and followed at our center. The study protocol was approved by the Ethical Committee, patients gave informed consent, and the study was conducted according to the Declaration of Helsinki. Primary AIHA was defined by hemolytic anemia and positive DAT, in the absence of underlying lymphoproliferative, infectious, autoimmune, or neoplastic diseases (15). Complete clinical examination, blood counts, hemolytic markers (reticulocytes, total and unconjugated bilirubin, LDH, and haptoglobin), and DAT were recorded at enrollment. Seventy-seven patients were classified as wAIHA (DAT+ for IgG only or IgG plus C3d) and 46 were classified as CAD (DAT+ for C3d only, with cold agglutinins titer ≥64), according to DAT and to the thermal characteristics of the autoantibody. Infectious and thrombotic complications were graded according to the Common terminology criteria CTCAE version 5.

### Cytokine genotyping

DNA was extracted from whole blood using the salting-out standard techniques. Cytokine genotyping by PCR-sequence specific primer (PCR-SSP) amplification was done for five cytokine genes SNPs: TNF-α, TGF-β1, IL-10, IL-6, and IFN-γ. The Cytokine Genotyping Tray (One Lambda, Canoga Park, CA) has been used to evaluate the TNF-α promoter -308G>A SNP (rs1800629); two TGF-β1 SNPs, codon 10 + 869C>T (rs1800470) and codon 25 + 915G>C (rs1800471); three IL-10 promoter SNPs, -1082A>G (rs1800896), -819 T>C (rs1800871), and -592A>C (rs1800872); the IL-6 promoter -174C>G (rs1800795); and IFN-γ +874T>A (rs2430561). These SNPs were selected on the basis of previous data in the literature on various autoimmune diseases, including AIHA (16–22). Allele and genotype frequency results were compared with those described in the Italian population by Poli et al. (2002) (23) and Uboldi de Capei et al. (2003) (24). The high-, intermediate-, and low-expression categories were defined according to available literature and the manufacturer instructions of the kit used.

### Cytokine serum levels

Peripheral blood serum samples were collected and the following cytokines have been tested in 64 patients using high-sensitivity ELISA kits: IL-6, IL-10, IFN-γ, TNF-α (High Sensitivity Elisa kits, eBioscience, Inc., Vienna Austria), and TGF-β (Immunological Sciences, Rome, Italy). Patient cytokine serum levels have been compared with median values of 40 healthy controls.

### Statistical analysis

Analysis of variance was performed by using mean, median, ranges, and standard errors. Student *t*-test has been used for continuous variables. For the comparison of categorical variables, Chi-squared or Fisher’s exact tests have been used appropriately.

## Results

### Clinical and laboratory characteristics of patients

**Table 1** shows the clinical and laboratory characteristics of the 123 patients studied. At diagnosis, wAIHA patients showed a more severe anemia compared to CAD ones (*p* < 0.0001) with higher LDH (*p* = 0.013) and absolute reticulocytes (*p* = 0.0004). During a median follow-up of 49 months (1–306), 52 patients required transfusions and 114 were treated (33 patients, 1 therapy line; 40 patients, 2 lines; and 41 patients, ≥3 lines). Nine patients did not require treatment (one wAIHA and eight CAD). The following complications were observed: 28 thrombotic events (18 in wAIHA and 10 in CAD), 52 infectious episodes ≥grade 3 (31 in wAIHA and 21 in CAD), and 18 patients (15%) died (7 wAIHA and 11 CAD), for causes not directly related to AIHA.

**Table 1:** Clinical and laboratory characteristics of patients.

	All AIHA (N=123)	wAIHA (N=77)	CAD (N=46)	P (wAIHA vs CAD)
Median Age at diagnosis (yrs, range)	63 (17-90)	62 (17-90)	66 (21-85)	NS
Gender (M/F)	51/72	33/44	18/28	NS
Hb (g/dL), median (range)	7.5 (3.5-14.4)	7.2 (3.5-14.4)	8.7 (4.9-13.3)	<0.0001
LDH (ULN), median (range)	1.8 (0.4-8.7)	2 (0.6-8.7)	1.3 (0.4-5.8)	0.013
Ret (x109/L), median (range)	186 (3-652)	214 (3-652)	137 (21-350)	0.0004
Unconjugated bilirubin (mg/L)	1.7 (0.1-8.5)	1.6 (0.1-8.5)	1.8 (0.3-4.9)	NS
Transfusion, N(%)	52 (42)	30 (39)	22 (48)	NS
Therapy lines				
0 line N (%)	9 (7)	1 (1.2)	8 (17)	0.003
1 line, N(%)	33 (27)	28 (36.4)	5 (11)	0.004
2 lines, N(%)	40 (33)	24 (31.2)	16 (35)	NS
≥3 lines, N(%)	41 (33)	24 (31.2)	17 (37)	NS
Complications, N(%)	80 (65)	49 (64)	31 (67)	NS
Infections, N(%)	52 (42)65	31 (40)63	21 (46)67	NS
Thrombosis, N(%)	28 (23)35	18 (23)37	10 (27)33	NS
Death, N(%)	18 (15)	7 (9)	11 (24)	0.05

LDH, lactate dehydrogenase; UNL, upper normal limit; AIHA, autoimmune hemolytic anemia; wAIHA, warm AIHA; CAD, cold agglutinin disease. NS= Not Significant

### Allele frequencies

We compared our frequency results with two different control cohorts, namely, control cohort 1 and control cohort 2 (23, 24). The allelic frequency of the TNF-α -308 G/A polymorphism was significantly lower in patients compared with both controls (**Supplementary Table 1**). In detail, the frequency of TNF-α -308G allele was 81% in all AIHAs and 77% in wAIHA, compared with 88% (control cohort 1) and 91% (control cohort 2). Non-significant differences between patients and controls were observed considering the other SNPs. No differences in allele distribution of any other cytokine SNPs (TGF-β1, IL-10, IL-6, and IFN-γ) were observed between wAIHA and CAD.

### Genotype frequency

**Table 2** shows the genotypic frequency of the cytokine gene SNPs. The frequency of the TNF-α -308G/A and the TGF-β gene codon 25 G/C genotypes were significantly lower in patients compared with controls. Specifically, the frequency of the TNF-α -308 GG genotype was 64% in all AIHAs and 58% in wAIHA, compared with 77% (control cohort 1) and 84% (control cohort 2). The frequency of the TGF-β gene codon 25 GG genotype was 77% in all AIHAs, 79% in wAIHA, and 74% in CAD versus 87% in controls.

**Table 2:** Genotypic frequency of the single nucleotide polymorphisms of cytokine genes.

	*Genotype*	*All AIHA* *(N=123)*	*wAIHA* *(N=77)*	*CAD* *(N=46)*	*Control cohort 1* *Poli et al. (N=363)* (23)	*Control cohort 2* *Uboldi et al. (N=140)* (24)
TNF-α -308 G/A	GG	79 (64) ^**,§^	45 (58) ^**,§^	34 (74)	277 (77)	118 (84)
	GA	42 (34)	30 (39)	12 (26)	79 (22)	19 (14)
	AA	2 (2)	2 (3)	0 (0)	3 (1)	3 (2)
TGF-β T/C codon 10	TT	51 (41)	33 (43)	18 (39)	ND	49 (32)
	TC	55 (45)	34 (44)	21 (46)	ND	63 (45)
	CC	17 (14)	10 (13)	7 (15)	ND	28 (23)
TGF-β C/G codon 25	CC	0 (0)	0 (0)	0 (0)	ND	4 (3)
	CG	28 (23)	16 (21)	12 (26)	ND	14 (10)
	GG	95 (77)^*^	61 (79)	34 (74)^*^	ND	122 (87)
IL10 -1082 A/G	AA	48 (39)	32 (42)	16 (35)	ND	48 (34)
	AG	62 (50)	39 (51)	23 (50)	ND	75 (54)
	GG	13 (11)	6 (8)	7 (15)	ND	17 (12)
IL10 -819 C/T	CC	58 (47)	39 (51)	19 (41)	ND	70 (50)
	CT	59 (48)	36 (47)	22 (50)	ND	62 (44)
	TT	6 (5)	2 (3)	4 (9)	ND	8 (6)
IL10 -592 A/C	AA	6 (5)	2 (3)	4 (9)	ND	8 (6)
	AC	59 (48)	36 (47)	22 (50)	ND	62 (44)
	CC	58 (48)	39 (52)	19 (42)	ND	70 (50)
IL6 -174 C/G	CC	16 (13)	7 (9)	9 (20)	40 (11)	13 (9)
	CG	58 (47)	39 (51)	19 (41)	142 (39)	70 (50)
	GG	49 (40)	31 (40)	18 (39)	180 (50)	57 (41)
IFN-γ +874 T/A	TT	25 (20)	14 (18)	11 (24)	77 (21)	ND
	TA	56 (46)	34 (44)	22 (48)	170 (47)	ND
	AA	42 (34)	29 (38)	13 (28)	116 (32)	ND

AIHA, autoimmune hemolytic anemia; wAIHA, warm AIHA; CAD, cold agglutinin disease.

Values are expressed as N(%). * = p<0.05 vs Uboldi et al ; **= p<0.001 vs Uboldi et al; § = p<0.05 vs Poli et al. ND=not determined

By investigating cytokine SNP genotypes associated with different gene expression levels (high versus intermediate versus low producers), the TNF-α high gene expression was significantly more frequent both in all AIHAs and in wAIHA patients compared with controls (36% and 41% versus 23% and 16%, respectively) (**Supplementary Table 2**). No other differences were observed considering other cytokine SNPs (TGF-β1, IL-10, IL-6, and IFN-γ).

### Cytokine SNP analysis according to clinical and hematological features

The frequency of TGF-β (T/T-G/G and T/C-G/G, 80% vs. 59%, *p* = 0.03) and IL-10 (GCC-GCC, 16% vs. 5%, *p* = 0.02) high gene expression level genotypes was higher in patients with more severe anemia (Hb ≤7.5 g/dL, i.e., median of all AIHA) versus the remaining AIHA population. Likewise, the frequency of IFN-γ high plus intermediate gene expression level genotypes (T/T plus T/A) was higher within the group with Hb levels ≤7.5 g/dL at diagnosis (74% vs. 57%, *p* = ns) (**Table 3**). The frequency of TGF-β high gene expression level genotypes was higher in patients with LDH greater than 1.8 xULN (i.e., the median of all AIHA, 80% vs. 58%, *p* = 0.05). Finally, considering treatment, TNF-α -308 high gene expression level genotypes were more frequent among CAD patients requiring three or more therapy lines (33% versus 20%). Likewise, TGF-β high gene expression level genotypes were more frequent in all multitreated AIHA patients (75% versus 60%) and CAD subgroup (76% versus 54%) (**Supplementary Table 3**).

**Table 3:** Gene expression levels of cytokine single nucleotide polymorphism in AIHA patients according to anemia and markers of hemolysis.

Cytokine Polymorphisms	Gene expression level	Corresponding genotypes	Hb (g/dL)^$^		LDH (ULN) ^$^		reticulocytes count (10^9^/L) ^$^		Bilirubin (mg/L) ^$^	
			≤7.5 (N=62)	>7.5 (N=61)	≤1.8 (N=57)	>1.8 (N=55)	≤181 (N=51)	>181 (N=50)	≤1.7 (N=50)	>1.7 (N=46)
TNF-α	High	AA; GA	22 (35)	22 (36)	20 (35)	19 (35)	17 (33)	18 (36)	15 (30)	18 (39)
-308 G/A	Low	GG	40 (65)	39 (64)	37 (65)	36 (65)	34 (67)	32 (64)	35 (70)	28 (61)
TGF−β	High	TT-GG ; C-GG	50 (80) *	36 (59)	33 (58)	44 (80) *	38 (71)	32 (64)	38 (72)	30 (65)
codon 10 C/T,	Intermediate	TC-GC ; CC-GG ; TT-GC	10 (16)	19 (31)	17 (30)	10 (18)	10 (20)	14 (28)	8 (16)	14 (30)
codon 25 G/C	Low	CC-GC ; CC-CC ; TT-CC ; TC-CC	2 (4)	6 (10)	7 (12)	1 (2)	3 (9)	4 (8)	6 (12)	2 (5)
IL10	High	GCC-GCC	10 (16) *	3 (5)	6 (11)	6 (11)	5 (10)	6 (12)	6 (12)	5 (11)
-1082 G/A, -819	Intermediate	GCC-ACC; GCC-ATA	24 (39)	37 (61)	32 (56)	23 (42)	21 (41)	25 (50)	22 (44)	22 (48)
C/T, -592 C/A	Low	ACC-ACC; ACC-ATA; ATA-ATA	28 (45)	21 (34)	19 (33)	26 (47)	25 (49)	19 (38)	22 (44)	19 (41)
IL6	High	GG; GC	55 (89)	52 (85)	49 (86)	49 (89)	46 (90)	45 (90)	43 (86)	41 (89)
-174 C/G	Low	CC	7 (11)	9 (15)	8 (14)	6 (11)	5 (10)	5 (10)	7 (14)	5 (11)
IFN-γ	High	TT	11 (18)	14 (23)	16 (29)	10 (18)	15 (29)	9 (18)	12 (24)	10 (22)
-874 T/A	Intermediate	TA	35 (56)*	21 (34)	23 (42)	28 (51)	20 (39)	26 (52)	21 (42)	24 (52)
	Low	AA	16 (26)	26 (43)	18 (33)	17 (31)	16 (32)	15 (30)	17 (34)	12 (26)

^$^ Hb =7.5 g/dL, LDH = 1.8 UNL, Reticulocytes count = 181 10^9^/L, bilirubin = 1.7 mg/L were the median of all AIHA patients at diagnosis.

Values are expressed as N (%). *=p<0.05 Hb>7.5 g/dL vs Hb≤7.5 g/dL.

### Cytokine serum levels

**Figure 1:**
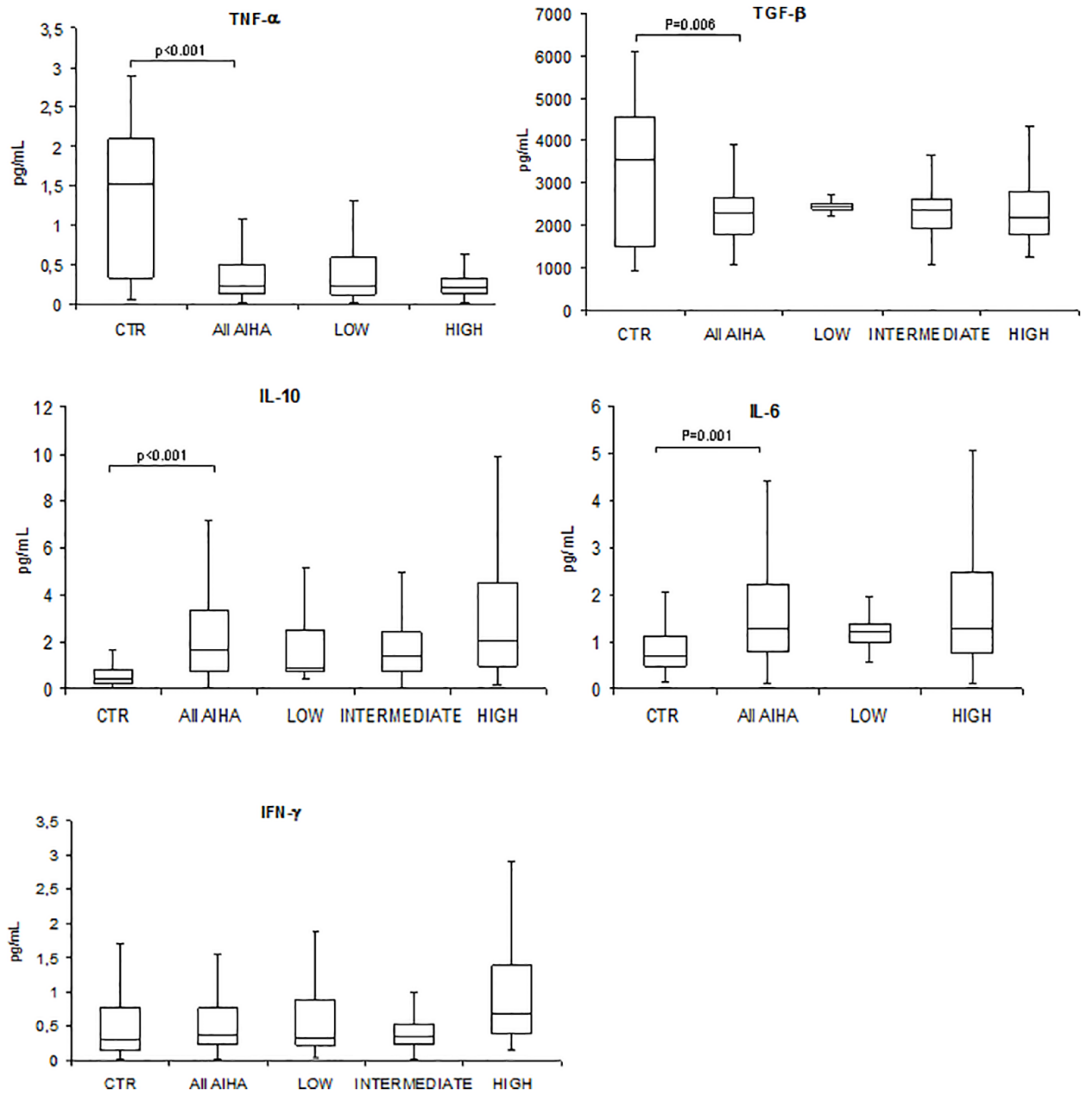
Cytokine serum levels in autoimmune hemolytic anemia patients (All AIHA) versus controls, and according to high/intermediate/low gene expression level. Tumor necrosis factor alpha (TNF-α), transforming growth factor beta (TGF-β), interleukin (IL)-10, IL-6, and interferon gamma (IFN-γ).

As shown in **Figure 1**, TNF-α and TGF-β serum levels were significantly lower in patients compared with controls (median 0.23 pg/mL, range 0.01–2.65 vs. 1.5, 0.1–2.9, *p* < 0.001 for TNF-α; 2,284 pg/mL, 1,076–5,908 vs. 3,531 pg/mL, 924–6086, *p* = 0.006 for TGF-β). Moreover, IL-10 and IL-6 serum levels were significantly higher in AIHA patients versus controls (median 1.67 pg/mL, 0.05–33.74 vs. 0.4 pg/mL, 0.02–2.7, *p* < 0.001 for IL-10; 1.26 pg/mL, 0.11–12.9 vs. 0.7 pg/mL, 0.2–4.7, *p* = 0.001 for IL-6). Finally, IFN-γ was slightly reduced versus controls (median 0.4 pg/mL, 0.01–11.40 vs. 0.7 pg/mL, 0.02–3.2), although not significantly. A slight association was found between gene expression genotypes and cytokine serum levels, i.e., high gene expression genotypes displayed higher cytokine levels: IL-6 (median 1.29 versus 1.26 pg/mL, not significant) and IFN-γ (median 0.7 versus 0.32 pg/mL, not significant). At variance, an inverse relationship between high gene expression genotypes and cytokine serum levels was found for TNF-α (median 0.21 versus 0.24 pg/mL, not significant), TGF-β (median 2,192 versus 2,425 pg/mL, not significant), and IL-10 (median 0.9 versus 2.08 pg/mL, not significant).

## Discussion

Here, we investigated several cytokine polymorphisms in AIHA and evaluated their relationship with cytokine serum levels and with disease severity. Overall, the allele frequency of some SNPs of TNF-α and the TNF-α and TGF-β genotypes were less frequently found in patients compared with controls (23, 24). Regarding TNF-α, our results are in agreement with those of Pavkovic et al., who found reduced G- and consequently increased A- alleles of TNF-α-308G/A in AIHA versus controls (16). At variance, no difference in genotype distribution, as compared to healthy controls, was reported by D’Abronzo et al. (25) who however investigated a small number of patients. Furthermore, in our study, the TNF-α genotype associated with high gene expression was more frequent in patients compared with controls, as well as in patients with more severe disease and generally in multitreated patients. It may be speculated that this genetic predisposition to a stronger inflammatory response may result in a greater immune dysregulation and in a relapsed/refractory disease. Alternatively, the association between TNF-α genotype and AIHA may be driven by the rarity of AA genotype. Surprisingly, TNF-α serum levels were found to be lower in AIHA than in controls, in agreement with several previous reports from our group (26–28). This apparent discrepancy may reflect the several incidental factors, such as overt and subclinical infections, inflammatory triggers, and treatments that may influence cytokine serum levels notwithstanding genetic background. Moreover, it may be assumed that cytokine serum levels represent the net result between production and utilization by cellular effectors. Thus, low levels may indicate a greater uptake and consumption in the context of a massive inflammatory/autoimmune process as observed in more severe disease. This has already been hypothesized for *in vitro* cultures assessing cytokine production and the effect of exogenous cytokines on autoantibody production: in the active disease, production of TGF-β was increased, and addition of TGF-β increased antibody production and binding to RBC. This leads to the speculation that overproduction of TGF-β may downregulate cellular responses (7). TGF-β is a pleiotropic cytokine regulating a wide variety of cell functions, with primarily anti-inflammatory, antimitotic, and profibrotic properties (29). Moreover, it could impair the generation of Th1 cells through an indirect IFN-γ-mediated mechanism and is a crucial inducer of T-helper 17 and Tregs. The role of TGF-β in autoimmunity is complex, depending on the phase of the disease and the main effector mechanisms involved (cellular versus humoral), but generally favors autoimmune phenomena. Here, we found that TGF-β high producer genotypes were more frequent in more hemolytic and multitreated AIHA patients, along with lower serum levels. As observed for TNF-α, the discrepancy between cytokine serum levels and high producer genotypes may be the result of cytokine utilization by effector cells.

For other cytokines, the picture is less clear-cut. Concerning IL-10, which is both a Th2 and anti-inflammatory cytokine, its serum levels were increased in patients versus controls, along with a greater frequency of high gene expression genotype in more severe cases. The increased IL-10 production may be an attempt to counteract the over-inflammatory autoimmune response, resulting in an excess free/unutilized cytokine in serum. Finally, serum levels of the Th2 and inflammatory cytokine IL-6 were found increased in patients consistently with the main humoral pathogenic mechanism of AIHA, although without a clear relationship with SNPs. The slight association found for IL-6 and IFN-γ cytokine serum levels and high gene expression genotype may possibly indicate cell autonomous activity of the cytokine, which contributes to an autocrine signaling. At variance, the absence of this association for TNF-α, TGF-β, and IL-10 suggests more complex regulatory pathways between genotype and phenotype.

In conclusion, the analysis of cytokine gene SNPs highlighted the role of the TNF-α− and TGF-β-mediated inflammatory response as a genetic background that may influence the severity and refractoriness of AIHA. Cytokine serum levels are one side of the coin, reflect the balance between production and utilization, and may be more influenced by incidental environmental factors. Overall, the results pinpoint the complexity of the cytokine network, where redundancy and pleiotropism of the various cytokines may generate opposite effects depending on the existing milieu.

## Data availability statement

The original contributions presented in the study are included in the article/**Supplementary Material**. Further inquiries can be directed to the corresponding author.

## Ethics statement

The studies involving humans were approved by Fondazione IRCCS Ca’ Granda Ospedale Maggiore Policlinico, Ethic Committee Milano Area 2. The studies were conducted in accordance with the local legislation and institutional requirements. Written informed consent for participation in this study was provided by the participants’ legal guardians/next of kin.

## Author contributions

BF and WB designed the study, followed patients, analyzed data, wrote the paper, and revised the paper for important intellectual content. AZ performed biological studies, collected and analyzed data, wrote the paper, and revised the paper for important intellectual content. LP followed patients and critically revised the manuscript. CV and AM collected samples and critically revised the manuscript. All authors contributed to the article and approved the submitted version.
