# Supplementary material for: Cytokine polymorphisms in patients with autoimmune hemolytic anemia
Source: Front Immunol. 2023 Nov 10;14:1221582. doi: 10.3389/fimmu.2023.1221582 (PMC10667680; doi:10.3389/fimmu.2023.1221582)
Supplement: Supplementary file 1 [file DataSheet_1.pdf]

Supplementary Table 1. Allelic frequency of the single nucleotide polymorphisms of cytokine genes

	Allele	*All AIHA (n=123)*	*wAIHA (n=77)*	*CAD (n=46)*	*Control cohort 1 Poli et al. (n=363)*[(23)]	*Control cohort 2 Uboldi et al. (n=140)*[(24)]
TNF-α -308 G/A	G	200 (81)[*]	120 (77) [**,§]	80 (87)	632 (88)	254 (91)
	A	46 (19)	34 (23)	12 (13)	88 (12)	26 (9)
TGF-β T/C codon 10	T	157 (64)	100 (65)	57 (62)	ND	144 (55)
	C	89 (36)	54 (35)	35 (38)	ND	126 (45)
TGF-β C/G codon 25	C	28 (11)	16 (10)	12 (13)	ND	22 (8)
	G	218 (89)	138 (90)	80 (87)	ND	258 (92)
IL10 -1082 A/G	A	159 (65)	106 (69)	53 (58)	458 (63)	170 (61)
	G	87 (35)	48 (31)	39 (42)	268 (37)	110 (39)
IL10 -819 C/T	C	175 (71)	114 (74)	61 (66)	514 (71)	202 (78)
	T	71 (29)	40 (26)	31 (34)	212 (29)	78 (28)
IL10 -592 C/A	G	175 (71)	114 (74)	61 (66)	514 (71)	202 (78)
	A	71 (29)	40 (26)	31 (34)	212 (29)	78 (28)
IL6 -174 C/G	C	90 (37)	53 (34)	37 (40)	210 (29)	96 (34)
	G	156 (63)	101 (66)	55 (60)	516 (71)	184 (66)
IFN-γ +874 T/A	T	108 (44)	62 (40)	46 (50)	326 (45)	ND
	A	138 (56)	92 (60)	46 (50)	398 (55)	ND

AIHA, autoimmune hemolytic anemia; wAIHA, warm AIHA; CAD, cold agglutinin disease.
Values are expressed as N%). * = p<0.05 vs Uboldi et al ; **= p=0.01 vs Uboldi et al ; § = p=0.05 vs Poli et al

Supplementary Table 2. Gene expression levels of cytokine single nucleotide polymorphism

Cytokine Polymorphisms	*Gene expression level*	*Corresponding genotypes*	*All AIHA (N=123)*	*wAIHA (N=77)*	*CAD (N=46)*	*Control cohort 1 Poli et al. (N=363)*[23]	*Control cohort 1 Uboldi et al. (N=140)* [24]
TNF-α	High	AA; GA	44 (36)[**,§]	32 (41) [**,§]	12 (26)	82 (23)	22 (16)
-308 G/A	Low	GG	79 (64)	45 (59)	34 (74)	277 (77)	118 (84)
TGF–β	High	TT-GG ; C-GG	86 (70)	54 (70)	32 (69)		98 (70)
codon 10 C/T,	Intermediate	TC-GC ; CC-GG ; TT-GC	29 (24)	20 (26)	9 (20)	ND	34 (24)
codon 25 G/C	Low	CC-GC ; CC-CC ; TT-CC ; TC-CC	8 (6)	3 (4)	5 (11)		8 (6)
IL10	High	GCC-GCC	13 (11)	6 (8)	7 (15)		17 (12)
-1082 G/A, -819 C/T,	Intermediate	GCC-ACC; GCC-ATA	61 (49)	38 (49)	23 (50)	ND	77 (55)
-592 C/A	Low	ACC-ACC; ACC-ATA; ATA-ATA	49 (40)	33 (43)	16 (35)		46 (33)
IL6	High	GG; GC	107 (87)	70 (91)	37 (80)	322 (89)	127 (91)
-174 C/G	Low	CC	16 (13)	7 (9)	9 (20)	40 (11)	13 (9)
IFN-γ	High	TT	25 (20)	14 (18)	11 (24)	77 (21)	
-874 T/A	Intermediate	TA	56 (46)	34 (44)	22 (48)	170 (47)	ND
	Low	AA	42 (34)	29 (38)	13 (28)	116 (32)	

AIHA, autoimmune hemolytic anemia; wAIHA, warm AIHA; CAD, cold agglutinin disease.
Values are expressed as N (%). **= p<0.001 vs Uboldi et al ; § = p<0.05 vs Poli et al

Supplementary Table 3. Cytokine gene single nucleotide polymorphism phenotypes in 123 AIHA patients according to number of therapy lines

Cytokine Polymorphisms	*Gene expression level*	*Corresponding genotypes*	*N of therapy lines*					
			All AIHA (N=123)		wAIHA (N=77)		CAD (N=46)	
			0/1 (N=42)	≥2 (N=81)	0/1 (N=29)	≥2 (N=48)	0/1 (N=13)	≥2 (N=33)
TNF-α -308 G/A	High	AA; GA	12 (36)	32 (40)	11 (39)	21 (44)	1 (20)	11 (33)
	Low	GG	30 (64)	49 (60)	18 (61)	27 (56)	12 (80)	22 (67)
TGF–β codon 10 C/T, codon 25 G/C	High	TT-GG ; C-GG	25 (60)	61 (75)	18 (64)	36 (73)	7 (54)	25 (76)
	Intermediate	TC-GC ; CC-GG ; TT-GC	12 (26)	18 (22)	9 (29)	11 (23)	3 (23)	7 (21)
	Low	CC-GC ; CC-CC ; TT-CC ; TC-CC	5 (14)	2 (3)	2 (7)	1 (4)	3 (23)	1 (3)
IL10 -1082 G/A, -819 C/T, -592 C/A	High	GCC-GCC	3 (7)	11 (14)	2 (7)	5 (10)	1 (8)	6 (19)
	Intermediate	GCC-ACC; GCC-ATA	23 (55)	37 (46)	16 (53)	21 (44)	7 (54)	16 (48)
	Low	ACC-ACC; ACC-ATA; ATA-ATA	16 (38)	33 (40)	11 (40)	22 (46)	5 (38)	11 (33)
IL6 -174 C/G	High	GG; GC	37 (88)	70 (86)	25 (86)	45 (94)	12 (92)	25 (76)
	Low	CC	5 (12)	11 (14)	4 (14)	3 (6)	1 (8)	8 (24)
IFN-γ -874 T/A	High	TT	13 (31)	13 (16)	8 (28)	6 (12)	5 (38)	7 (21)
	Intermediate	TA	16 (38)	40 (49)	10 (32)	24 (50)	6 (46)	16 (48)
	Low	AA	13 (31)	28 (35)	11 (40)	18 (38)	2 (16)	10 (31)

AIHA, autoimmune hemolytic anemia; wAIHA, warm AIHA; CAD, cold agglutinin disease.
